# Use of Reflective Tape to Detect Ultrasound Transducer Movement: A Validation Study

**DOI:** 10.3390/life11020104

**Published:** 2021-01-30

**Authors:** Lisa Mohr, Lutz Vogt, Jan Wilke

**Affiliations:** Department of Sports Medicine, Goethe University Frankfurt, 60323 Frankfurt/Main, Germany; l.vogt@sport.uni-frankfurt.de (L.V.); wilke@sport.uni-frankfurt.de (J.W.)

**Keywords:** ultrasound, reflective tape, transducer movement

## Abstract

During dynamic ultrasound assessments, unintended transducer movement over the skin needs to be prevented as it may bias the results. The present study investigated the validity of two methods quantifying transducer motion. An ultrasound transducer was moved on a pre-specified 3 cm distance over the semitendinosus muscle of eleven adults (35.8 ± 9.8 years), stopping briefly at intervals of 0.5 cm. Transducer motion was quantified (1) measuring the 2-D displacement of the shadow produced by reflective tape (RT) attached to the skin and (2) using a marker-based, three-dimensional movement analysis system (MAS). Differences between methods were detected with Wilcoxon tests; associations were checked by means of intraclass correlation coefficients (ICC 3.1) and Bland–Altman plots. Values for RT (r = 0.57, *p* < 0.001) and MAS (r = 0.19, *p* = 0.002) were significantly higher than true distances (TD). Strong correlations were found between RT and TD (ICC: 0.98, *p* < 0.001), MAS and TD (ICC: 0.95, *p* < 0.001), and MAS and RT (ICC: 0.97, *p* < 0.001). Bland–Altman plots showed narrow limits of agreement for both RT (−0.49 to 0.13 cm) and MAS (−0.49 to 0.34 cm) versus TD. RT and MAS are valid methods to quantify US transducer movement. In view of its low costs and complexity, RT can particularly be recommended for application in research and clinical practice.

## 1. Introduction

Ultrasound (US) imaging is a popular method in both medical examination and scientific research. Besides visualizing internal organs, health professionals typically apply US to evaluate elements of the musculoskeletal system, i.e., the morphology [1,2,3] and mechanical behaviour of the soft tissue [4]. With regard to sports medical screenings, assessments can be static or dynamic. Static measurements without movement of the targeted structure, inter alia, include the quantification of muscle thickness [1,5,6,7,8,9] or the search for tissue damage following injury [3,9,10,11]. Dynamic measurements require motion of the studied object. Typical examples are the investigation of nerve sliding [12], fascial movement [13,14] or muscle contraction [15,16,17,18].

In most dynamic US assessments, it is paramount to prevent unintended movement of the US transducer, as this would create artefacts. It has been demonstrated that even small changes in the orientation of the US transducer may represent a potential source of error, biasing the results of the investigation [18,19,20,21]. One example for measurement inaccuracies due to probe movement may be the visualization of a muscle during the different phases of a stretch. Here, contrary to static assessments, where it is relatively easy to keep the probe exactly in the same place, the lengthening of the soft tissue may cause unintended transducer motion due to the glide-friendly nature of the US gel. If the produced video would now be analysed for parameters such as thickness, it may not show the exact same places at the beginning and end of the stretch. As a consequence, possible differences could relate to both the actual stretch of the tissue and the fact that the transducer moved (e.g., showing the tissue one centimetre to the left).

A variety of strategies have been applied to control track movements during US measurements. Some included complex movement analysis systems (e.g., Vicon [22] or Qualisys [19]), which, however, require high time efforts and considerable financial resources. Another approach is the use of acoustically reflective markers (e.g., micropore tape) attached to the skin [7]. As they are visible in the US image and as their displacement upon transducer movement can be quantified, they may represent an easy-to-handle and affordable alternative to more complex methods. However, to date, there is no data describing the value of the different approaches in detecting US transducer movement. The present study therefore aimed to investigate the validity of transducer movement assessments using (a) reflective tape and (b) a three-dimensional motion analysis system.

## 2. Materials and Methods

### 2.1. Design and Ethics

A cross-sectional study was performed in May and June 2020. It was approved by the Department 05 Ethics Committee of Goethe-University Frankfurt (2020–30) and conducted according to the Declaration of Helsinki as well as the guidelines of Good Clinical Practice. All enrolled participants provided written informed consent.

### 2.2. Sample

A total of 11 healthy adult participants (age = 35.8 ± 9.8 years; height =176.0 ± 0.1 cm; weight = 76.3 ± 14.6 kg; BMI 24.3 ± 4.0; 4 women, 7 men) were recruited using poster advertising and word of mouth. Exclusion criteria were (1) presence of skin allergies not allowing US screenings, (2) history of injury or overuse disorders in the lower limb, (3) presence of severe chronic diseases, and (4) pregnancy or nursing period.

### 2.3. Examination

**Figure 1:**
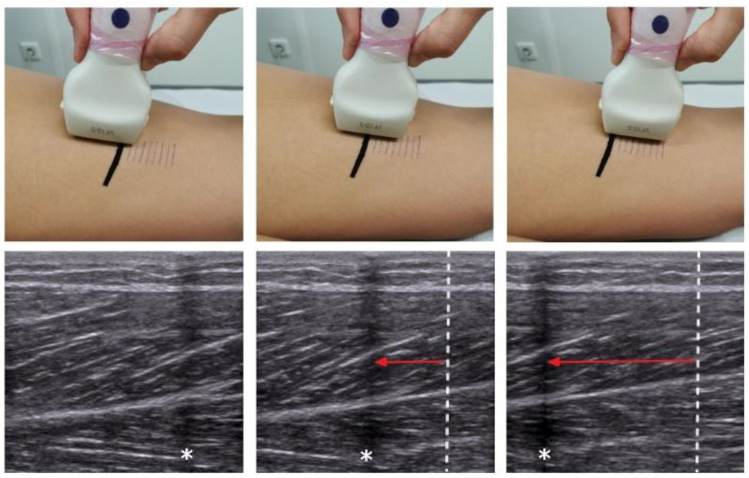
Position and movement of the ultrasound (US) transducer on the skin (**top**) and the resulting US images (**bottom**). Initially (**left**), the shadow of the reflective tape (asterisk) can be seen at the right border. Upon probe movement (**middle** and **right**), this shadow is displaced relative to the original starting position (dotted line). The distance (red arrow) is quantified using software.

All measurements were conducted in the same room and at constant temperature and daytime. Participants adopted a standardized prone position on a treatment table. They were asked not to move and to remain relaxed during the following examination. Firstly, using palpation and US, the semitendinosus muscle was identified as described by Balius et al. [23]. On the muscle belly, a distance of 3 cm, divided into sections of 0.5 cm was marked with a pen (Figure 1). Reflective micropore tape (producing a visible shadow in the US image) was placed at the starting point of the 3 cm section. A trained investigator then longitudinally moved the 5 cm linear array transducer (frequency: 8 MHz, amplification: 30 dB) of a high-resolution US system (Siemens Acuson X300, Siemens Medical Solutions USA, Inc., Mountain View, CA, USA) over the 3 cm distance, stopping at each 0.5 cm section for one second (Figure 1). A US video was recorded (depth of the ultrasound image: 3.5 cm) throughout the examination, which was performed in both legs.

### 2.4. Outcomes

**Figure 2:**
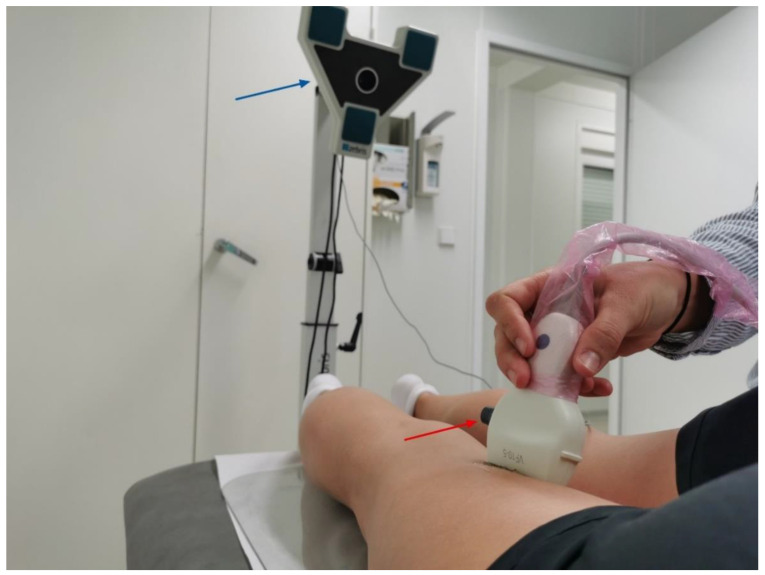
Ultrasound-based, three-dimensional movement analysis system. The photo shows the microphone of the device (blue arrow) and a marker (red arrow) attached to the transducer. The marker constantly sends an acoustical signal which is recorded by the microphone. As the transducer (and with this, the marker) is moved over the marked 3 cm distance on the skin, the travel time changes, which indicates probe (marker) movement.

Movement of the transducer was quantified in two ways. Firstly, the 2D US recordings were used to quantify the reflective tape’s (RT) displacement, which is a surrogate of probe motion. For analysis, the captured video was exported to the software ImageJ (Rasband, W.S., ImageJ, U.S. National Institutes of Health, Bethesda, MA, USA; [7,14,24]). At the starting position (0 cm) as well as at each of the pre-defined stopping points (0.5, 1, 1.5, 2, 2.5, 3 cm, true distance/TD), the video was frozen and three equidistant regions of interest (ROI, circular diameter 3 mm) were selected within the centre of the respective shadows produced by the reflective tape. The MTrackJ plugin [25] of the software quantifies the length of the track between the ROIs in the starting position and the respective distances (Figure 1). The required time for analysis was approximately 5 min. Pilot measurements with two repeated assessments demonstrated the described approach (detection of probe movement using reflective tape and ImageJ) to be highly reliable (intraclass correlation coefficient of reliability, ICR = 0.99). Secondly, an ultrasound-based, three-dimensional movement analysis system (MAS, Zebris CMS 10-6-2, Zebris Medical GmbH, Isny, Germany) was used to calculate probe movement based on tracking of acoustic markers (Figure 2). The MAS measures the travel time of the ultrasonic impulses sent from the sensors attached to an object of interest (in this case the US transducer) to a stationary microphone. If sensors are approached to or moved away from the microphone, the travel time of the signal shortens or lengthens accordingly, which indicates movement of the object of interest. Using triangulation, the spatial 3D-coordinatres of the markers (sampling rate 100 Hz) are determined by the software (analysing time approximately 15 min). The device collects external kinematic data with an accuracy of >0.6 mm [26]; the inter- as well as intra-rater reliability have been described as being good to excellent (r between 0.84 and 0.96 [27]).

For both ways of assessment (RT and MAS), we calculated the obtained mean values at each stopping point of the 3 cm distance (0.5, 1, 1.5, 2, 2.5, 3 cm). Differences between methods were examined using Wilcoxon tests for dependent samples. To judge agreement, Bland–Altman plots for multiple measurements were constructed. In addition, associations were examined by means of the intraclass correlation coefficient (ICC 3.1). Resulting effect sizes were interpreted as negligible (0.0 to 0.3), low (0.3 to 0.5), moderate (0.5 to 0.7), high (0.7 to 0.9), or very high (0.9 to 1.0) according to Koo und Li [28]. The level of statistical significance was set to α < 0.05. A Kolmogorov–Smirnov test was used to check the data for normal distribution. All calculations were performed with BiAs 11.12 (Goethe University, Frankfurt, Germany).

## 3. Results

**Figure 3:**
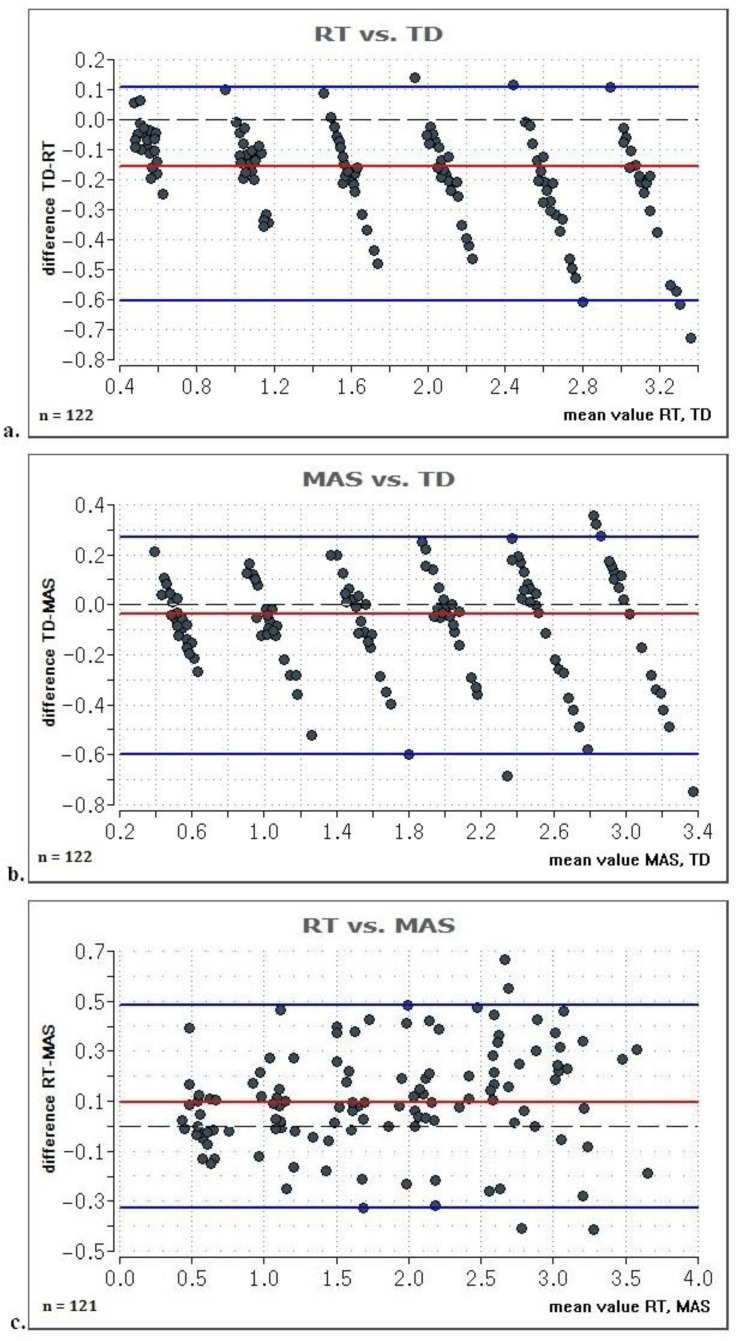
Bland–Altman-plots displaying the individual values assessed with the two values (**a**) RT vs. TD; (**b**) MAS vs. TD; (**c**) RT vs. MAS. RT = reflective tape, MAS = movement analysis system, TD = true distance, blue line = 95% limits of agreement (LoA); red line: mean value of the difference; dashed line: optimal zero line in case of agreement.

Both MAS and RT showed significantly higher values than the reference (RT vs. TD: r = 0.57, *p* < 0.001, MAS vs. TD: 0.19, *p* = 0.002, Table 1). However, strong correlations were found between MAS and TD (ICC = 0.96, 95% CI: 0.95 to 0.97 *p* < 0.001), RT and TD (ICC = 0.98, 95% CI: 0.97 to 0.98), *p* < 0.001) as well as MAS and RT (ICC = 0.97, 95% CI: 0.96 to 0.98, *p* < 0.001). Bland–Altman plots revealed narrow limits of agreement for both RT (−0.49 to 0.13 cm) and MAS (−0.49 to 0.34) vs. TD (Figure 3).

## 4. Discussion

To the best of our knowledge, this study is the first to examine the validity of two methods aiming to quantify transducer motion during US-based assessments of soft tissue. Our main finding is that both tracking the displacement of reflective tape in the US image and the use of an MAS are generally valid methods for the measurement of probe movement over the skin.

Controlling US transducer motion has several applications in both research and clinical practice. In non-specific arm pain or after whiplash injury, nerve sliding may be reduced [29]. Similarly, patients with non-specific low back pain have been demonstrated to exhibit lower shear mobility in the lumbar fascia than healthy controls [30] and individuals with Achilles tendinopathy display decreases in intra-tendinous tissue displacement [31]. In all these examples, US measurements were taken during active or passive benchmark movements potentially causing transducer motion, which needs to be corrected.

Interestingly, the displacement distances measured with the two methods always exceeded the TD. With differences averaging 9–17%, the deviation was higher for RT than for MAS (2 to 11%). Although the collected data systematically overestimate the actual values, the MAS, hence, was slightly more precise than RT. As a consequence, particularly if smallest displacements are to be detected, a movement analysis system with markers may be preferable. However, using RT may still represent a valuable option in some cases. When selecting diagnostic methods and outcomes, two factors need to be thoroughly balanced. Effort, on the one hand, depends on time as well as financial or personal resources. Psychometric effectiveness, on the other hand, is governed by a method’s inert or associated precision, validity, and reliability [32]. In many, albeit not all cases, it has been shown that both factors are correlated, meaning that the more efficient or gold standard methods are more expensive and more complex than simple and affordable methods [33,34,35]. Arguably, this is why many research facilities are equipped with expansive laboratories and a variety of devices. The MAS used in this study has its main applications in research, above all for measuring joint range of motion (e.g., [36,37]). In clinical practice, measurements have to be straightforward, affordable, and fast. In only 20 min, representing the average duration of a visit, physicians have to perform anamneses, conduct own assessments, and apply or prescribe treatments or drugs [38]. Similarly, health professionals in sports do only have seconds or minutes to screen injured athletes on the pitch or court. Using an MAS for tracking transducer movement against this background has three potential caveats. Firstly, as said, practitioners and smaller research facilities may not have the required financial resources. Secondly, preparing and performing measurements is far more time-consuming than using a stripe of tape: Analysing the MAS data takes approximately 15 min which adds to the 5–10 min needed to prepare a measurement (set up, adjust, calibrate, etc.). In contrast, RT requires only seconds to minutes and, if the visual evaluation of the shadow’s movement in the live US image does not suffice, an additional 5 min for quantification in ImageJ. Finally, during dynamic measurements, the markers attached to the US transducer may be hidden by other body parts—for example, when examining the gastrocnemius muscle during a squat or lunge. Hence, in all these cases, and if not aiming for the highest possible precision, applying RT seems to represent an excellent approach to increase the validity of findings. This has particular relevance because portable 2D-US devices have become more and more popular in sporting events [10] and because US is increasingly used in rehabilitation (a) for assessments and (b) as a feedback device during exercise.

Some limitations of our study need to be discussed. During the measurements, the US transducer was stabilized by hand, taking great care to maintain its position. Although this approach proved workable, very small probe movements in other dimensions may still have occurred. Future studies should consider fixing the transducer to the skin, e.g., by means of foam templates. With regard to the magnitude of potential probe displacement, we chose to examine distances between five mm and three cm because these seem realistic and relevant movements occurring in clinical practice. However, in some studies, even smaller displacements need to be detected, and hence, future research may aim to expand our findings in this sense. Due to manual probe handling, it is also possible that the pre-specified distances (0.5 to 3 cm) have not been reached exactly. Besides an inert imprecision of the MAS and the RT measurements, this could also explain the systematically higher values produced by both approaches when compared to the TD. In upcoming validation trials, it would be of interest to combine the use of tissue phantoms and foam templates to delineate the relative contribution of both potential sources of error. Finally, another issue relates to the number of measurements. We performed only one repetition because both the use of the MAS and the RT has been demonstrated to be highly reliable [14,27]. However, averaging repeated measures may still have increased the accuracy of our comparison.

## 5. Conclusions

Using reflective tape as a reference in the quantification of 2D US transducer movement is an easy-to-handle, affordable, and valid alternative to more complex methods such as three-dimensional movement analysing systems. However, both methods examined in this study tended to overestimate the true values.

## Figures and Tables

**Table 1:** Mean distances measured with reflective tape (RT) and movement analysis system (MAS) at the respective stopping points as well as absolute and relative differences between the used methods. TD = true distance.

TD [cm]	RT [cm]	MAS [cm]	∆TD-RT [cm]	∆TD-RT [%]	∆TD-MAS [cm]	∆TD-MAS [%]
0.5	0.582	0.557	0.082	16.5	0.057	11.4
1	1.150	1.088	0.150	15.0	0.088	8.8
1.5	1.679	1.583	0.179	11.9	0.083	5.5
2	2.177	2.069	0.177	8.8	0.069	3.5
2.5	2.751	2.572	0.251	10.0	0.072	2.9
3	3.258	3.062	0.258	8.6	0.062	2.1

## Data Availability

Data can be made available by the author upon request
